# Supplementary material for: Neonatal outcomes and resuscitation practices following the addition of heart rate-guidance to basic resuscitation
Source: PLoS One. 2025 Jan 29;20(1):e0317199. doi: 10.1371/journal.pone.0317199 (PMC11778765; doi:10.1371/journal.pone.0317199)
Supplement: Attachment — Submitted filename: Response to Reviewers PLOS One 11-20-24.docx [file pone.0317199.s001.docx]

November 20, 2024

Dr. Ribka Amsalu
Academic Editor
PLOS ONE

Dear Dr. Amsalu,

Thank you for your further consideration of our manuscript titled “*Neonatal outcomes and resuscitation practices following the addition of heart rate-guidance to basic resuscitation” (PONE-D-11287).* We thank you and the reviewers for their helpful comments, and respectfully submit the final revised manuscript. Below is a point-by-point response to the reviewers’ comments.

Sincerely,

Jackie K. Patterson, MD, MPH

Associate Professor of Pediatrics

Division of Neonatal-Perinatal Medicine

University of North Carolina at Chapel Hill

**Response to Reviewer 1 Concerns**

*Methods*

1) The primary outcome measure (effective breathing at 3 minutes after birth) (Line 91) doesn’t seem to match the stated primary objective (evaluating impact on stillbirth misclassification) (Line 101). Wouldn’t it be best for the primary objective to focus on the primary outcome measure? The current primary outcome measure is more aligned with the stated secondary objective (Line 103). Would you like to switch the stated primary and secondary objectives so they better match the primary outcome measure?

**Response:**
The study we describe in this manuscript is a sub-study of the primary trial; we realize from your comment above that it was confusing to report the objectives per the primary trial. As such, we have simplified our reference to the primary trial and focused on the primary objective of the study described in this manuscript. The methods starting on line 104 of the revised manuscript now reads as follows:

“We conducted a pre-post interventional trial in three urban health facilities in the DRC to evaluate the impact of use of continuous electronic HR monitoring during resuscitation (ClinicalTrials.gov Identifier: NCT03799861). The study design and participants for this trial have been previously described. The primary objective of this study was to evaluate the impact of resuscitation training in HBB (control group) compared to HR-guided basic resuscitation (intervention group) on effective breathing at three minutes after birth.” (Lines 109-110)

2) In this particular study, which included interim provider practice (~monthly) and additional training and simulation during the HR training, I have some concern with the pre-post study design being able to distinguish the impact from NeoBeat (discussed below). The lack of randomization may introduce biases. A cluster-randomized trial would strengthen the study's design and is proposed by the authors in their Conclusion. There is no mention of blinding, which could bias the outcomes assessed by the research staff. This could be mentioned in one’s Limitations paragraph.

**Response:**

We added these concerns to the limitations paragraph starting on line 512 and reading as follows:

“Most importantly, the pre/post study design and lack of randomization does not allow us to distinguish the relative contribution of HR-guided resuscitation versus on-going simulation training to the change in outcomes. Neither participants nor research staff were blinded to the intervention.” (Lines 512-513)

3) Line 116: I understand it may be described in other publications, but it would likely be useful to readers if the authors provide a brief description of the NeoBeat device (e.g., reusable, hands-free device placed on the chest of the newborn, etc.).

**Response:**

We have added this content on line 129 of the revised manuscript, which now reads:

“NeoBeat is a re-usable, C-shaped device that measures HR via dry-electrode technology and is easily placed around the thorax of a newborn by a single provider.” (Lines 129-131)

4) Line 117: The authors mention the first phase of the trial included HBB training and that “no guidance on use of HR data to inform resuscitation steps was provided during this control phase.” While I agree that much of the focus of HBB is on newborn breathing rather than HR, the HBB training materials also mention HR evaluation repeatedly — and HR is the focus of the last part of the Action Plan, in particular, beginning on Page 38 of the HBB2 Provider Manual. I would suggest the authors please reword this sentence.

**Response:**

We have clarified that the guidance provided on HR during HBB training conformed to the standard HBB materials. The methods now read:

“Guidance on use of HR to inform resuscitation steps was provided based on the standard HBB curriculum, which emphasizes evaluation of HR after improving ventilation.” (Lines 131-133)

5) Line 132: As the article is written for an international audience, I would suggest the authors provide a reference for NRP and/or cite it as the training program of the AAP.

**Response:**

We added a reference and clarified the program is the AAP’s in the text.

6) Line 171: Minor comment, but “data” is technically a plural noun so its verb (“was”) should be plural.

**Response:**

Corrected.

7) Line 188: Instead of including “total stillbirths,” shouldn’t the definition of perinatal mortality be “fresh stillbirths + death before discharge”? I don’t believe macerated stillbirths are included in perinatal mortality.

**Response:**

Perinatal mortality is variably defined in the literature, and sometimes includes all stillbirths but other times is restricted to fresh stillbirths. In the primary trial in which we conducted this sub-study, we elected to use total stillbirths as the primary outcome due to concern for the poor accuracy of clinical exam in determining timing of stillbirth. In keeping with the primary trial, we maintained the same definition of perinatal mortality for this sub-study. We have added this explanation to the methods, which now reads as follows:

“While we did not anticipate HR-guided basic resuscitation to change the outcome of antepartum stillbirths, we elected to include all stillbirths in this definition of perinatal mortality due to concern for the inaccuracy of clinical exam in distinguishing the timing of stillbirth; this decision is also in keeping with the primary trial in which this sub-study was conducted.” (Lines 213-217)

8) Line 196: The authors identify misclassification of a stillborn case includes the presence of a documented HR. However, shouldn’t they be including any sign of life (e.g., a respiratory gasp, any movement, etc.) and not just a documented HR?

**Response:**

We did not collect data on other signs of life besides breathing. As such, to determine misclassification, we relied solely on objective HR data. If the neonate was classified by the provider as stillborn and there was no HR registered by NeoBeat, we assumed their classification was correct. If the neonate was classified by the provider as stillborn, but NeoBeat registered a HR, we assumed the neonate had been misclassified. To clarify, we deleted the phrase about observational data in this section of the methods. It now reads:

**“**In post-hoc analysis, we conducted expert review of all stillborn cases for which we had HR data to determine the rate of misclassification based on presence of a documented HR as previously described.” (Lines 222-224)

*Results*

9) Not essential, but additional subgroup analyses (e.g., based on gestational age) could provide deeper insights into which populations benefit most from HR-guided resuscitation.

**Response:**

Given your concerns about teasing out whether changes in practice were due to on-going simulation training versus HR-guided resuscitation, we have elected not to pursue subgroup analyses as we will be unable to draw any firm conclusions from this additional exploration.

10) Lines 217-218: Please clarify in the text that this is the *post* OSCE data, as you did in Table 1.

**Response:**

Clarified.

11) Line 219: As above, should be “Data were”

**Response:**

Corrected.

12) Table 2: Could the authors add an additional column that includes p-values comparing HBB to HR-guided basic resuscitation?

**Response:**

Added.

13) Table 3: Just a note that there appears to be a fair amount of missing data (e.g., 35.9% of HBB data). This could be briefly mentioned in the Limitations.

**Response:**
Added as a sentence in the limitations as follows:

“More than one third of 5-minute Apgar scores were missing during the control phase.” (Line 520-521)

*Discussion*

14) Important: Lines 266-268: The authors report that, in addition to decreasing the time to BMV post-intervention, participants also reduced the time to some of the initial HBB steps. These initial resuscitation steps aren’t informed by HR, yet they improved, nonetheless. This suggests to me that these early resuscitation steps and, therefore, at least in part, BMV likely improved because of the interim provider practice and the additional training/practice during the HR training. It’s, therefore, unfortunately tough to say whether any of the improvements in the initial HBB steps or, more importantly, in BMV were attributable to the NeoBeat. I recognize that the authors may be tangentially alluding to this in their discussion of “time-dependent improvement in resuscitation practices” (Lines 313-319). However, I think the issue goes beyond just passive improvement with time; in the post-intervention period (i.e., HR-guidance phase), the providers not only had ~monthly HBB practice sessions but had also received additional HBB training and simulation during the HR training itself. (While I imagine the HR training had a particular focus on the HR portion of the HR-adapted HBB Action Plan, certainly providers were also refreshed to the other portions of the Action Plan, etc. during this HR training.) Therefore, I fear it is quite difficult to ascertain what role NeoBeat played in any improvements in provider skills, correct? Please correct me if I’m missing something. Otherwise, I think the Discussion, Conclusion, and Abstract need to be written in that light, and one couldn’t claim, for example, “HR-guided basic resuscitation resulted in earlier ventilation” (Lines 286-287), “Access to continuous HR data improved time to response…” (Lines 342-344), and other statements in the Discussion, Conclusion, and Abstract.

**Response:**

Thanks for this very important comment. We agree that the study design does not allow us to tease out the effect of refresher training and on-going LDHF practice from HR-guided resuscitation. Accordingly, we have tempered our language in the abstract, discussion and conclusion. See changes on lines 47-48, 292-295, 310-311, 339-352, 356-358, and 382-384.

15) Line 298: Do the authors know whether the rates of delayed cord clamping were roughly similar during the pre vs post phases? DCC may play a role in ‘time to BMV’ if there’s a delay in initially recognizing a non-vigorous newborn and cutting the cord to facilitate BMV. However, if DCC rates were higher in the post-intervention period (which may have occurred with more recent newborn practices), the improvements in time to BMV may be even more noteworthy.

**Response:**

There was no difference in the time to cord clamping between HBB and HR-guided resuscitation. While the HR-guided resuscitation epoch was not associated with earlier cord clamping, we hypothesize that once neonates were at the warmer, providers were quicker to move on to BMV when they did not respond to suctioning. We have added data on cord clamping to Table 5.

16) Limitations: In addition to the limitation comments above, several confounding variables could influence the results of this study and could be mentioned and/or discussed in the Limitations paragraph, including any pre/post differences in GA, BW, maternal factors, seasonality, concomitant changes in clinical practices, etc.

**Response:**

We have added a sentence regarding confounding variables to the limitations paragraph as follows:

“Several confounding variables may have influenced our results including pre/post differences in baseline maternal, intrapartum and neonatal characteristics, and systematic changes in clinical practice.” (Lines 513-516)

*Conclusion*

17) Lines 340-342: As discussed above, I have concern with concluding “Access to continuous HR data improved time to response…”

**Response:**

We have tempered the first few sentences of the conclusion, which now read as follows:

“During HR-guided resuscitation, providers were more efficient in responding to non-breathing neonates; however, significant gaps in quality care remain. Our results support the feasibility and potential benefit of integrated HR monitoring in basic resuscitation.” (Lines 538-541)

*Acknowledgements*

18) Minor point, but I believe “implement” should be “implemented”

**Response:**

Corrected.

**Response to Reviewer 2 Concerns (Statistical Review)**

1) Little is said about the statistical model that has been implemented for the analysis of the primary outcome and such little is also quite obscure. For example, the abstract says "using a method of least squares to fit general linear models". I guess the authors are dealing with generalized (not general) linear models, and these models are fitted by maximum likelihood methods (not least squares). Then lines 198-201 say "We used a method of least squares to fit general linear models using an identity link and binomial distribution assumption to evaluate whether HR-guided basic resuscitation results in an increased probability that infants breathe effectively within three minutes compared to HBB". An identity link function does not make sense under a binomial assumption. Maybe the authors are just misinterpreting statistical terminology, but correct terminology allows to know what exactly has been done, facilitating results reproducibility and interpretation.

**Response:**

We were incorrect in stating this was an identity link; we used a canonical logit link for this analysis. For both primary and secondary outcomes, the models used to obtain Adjusted Relative Risks and Beta-estimates are all generalized linear models using maximum likelihood estimation. The models all use a canonical logit link function and now control for (1) epoch (HBB vs. HR-Guided groups), (2) facility, (3) gestational age and (4) maternal age. We have edited the statement in the abstract as follows:

“We evaluated our primary outcome of effective breathing at three minutes after birth among newborns not breathing well at 30 seconds after birth employing generalized linear models using maximum likelihood estimation.” (Lines 34-37)

We have also edited the statement in the methods as follows:

“For both primary and secondary outcomes, we employed generalized linear models using maximum likelihood estimation. The models all used a canonical logit link function and controlled for epoch (HBB vs HR-guided groups), facility, gestational age, and maternal age. All continuous outcomes were evaluated using linear regression and all categorical outcomes were dichotomized and evaluated using logistic regression.” (Lines 210-218)

2) Although it is not entirely clear (see major point no 1), I guess the authors are fitting a logistic regression model with a canonical logit link for the analysis of the primary outcome. The results are displayed in Table 4 (first row) but they are "adjusted for facility". What does it mean? Is this the "treatment" effect after removing facility-specific differences? In this case, the estimate of beta should be provided along with the facility differences (are they significant?). More generally, it looks like only facility was included as a confounder. What about all the other variables that are summarized in Table 2? Results can be severely biased if these confounders are significantly correlated with the response variable. The coefficient beta should be estimated along with all these confounders.

**Response:**

See response to #1 above. To account for potential variations in resuscitation skills and resource availability across different facilities, we included facility as a control variable in our analysis. This allowed us to isolate the specific impact of HBB and HR-guided training on outcomes while accounting for potential differences in resuscitation skills and facility-level resources that might influence results.

Per your comment above, we have added gestational age and maternal age as control variables and updated our methods (see lines 210-218) and results accordingly.

3) Nothing is said about the statistical models that have been estimated for the analysis of the secondary outcomes. Some of these outcomes are in the form of time up to an event (e.g. time to effective breathing or time to HR≥100 bpm), hence hazard-based regression models are appropriate. Other outcomes are in the form of a binary event (HR≥100 bpm at three minutes after birth or death before discharge) and hence logistic regression is appropriate. The Apgar index is an integer between 0 and 10 and requires a binomial regression or, less rigorously, a linear regression model if the index is approximately normally distributed. Without this information, the estimated treatment effect cannot be interpreted.

**Response:**

We used a generalized linear model for all secondary outcomes that were continuous variables. For non-linear secondary outcomes such as Apgar score, we dichotomized to a binary variable (e.g., was Apgar score ≤5 at 1 minute?) and then used a logistic regression model.

We edited the statement in the methods as follows:

“For both primary and secondary outcomes, we employed generalized linear models using maximum likelihood estimation. The models all used a canonical logit link function and controlled for epoch (HBB vs HR-guided groups), facility, gestational age, and maternal age. All continuous outcomes were evaluated using linear regression and all categorical outcomes were dichotomized and evaluated using logistic regression.” (Lines 210-218)

4) It seems that the confounders of Table 2 have been ignored in the analysis of the secondary outcomes. Results can be severely biased if these confounders are significantly correlated with the response variable. Relative risks and regression coefficients should be estimated along with all these confounders.

**Response:**

We added gestational age and maternal age as control variables in addition to facility and updated our methods (see lines 210-218) and results accordingly.
